# On Cantharidian and Its Relation to Spanish Flies

**Published:** 1856-10

**Authors:** 


*On Cantharidian, and its Relation to Spanish Flies.* By Dr. Schroff. (Reitsch. der k. k. Gesellsch. du Aerzte zu Wien. July and August 1855.)

Comparative experiments were made upon rabbits with the cantharidian and Spanish flies, and one comparative experiment by M. C. Heinrich, who took at one time 10 drops of a strong tincture of cantharides, prepared by himself from fresh undried flies; and at another time, 0.01 gramme of cantharidin. It is clear from these observations that the cantharidin is the irrtating principle of the flies, as it not only produced gastro-enteritis, but, after absorption, also proved irritant to the urinary organs. One interesting result obtained by Heinrich is, that the cantharidin, although producing inflammation along the whole digestive tube, and in the urinary organs, failed to produce any excitement whatever of the sexual system, while the latter was a marked effect of the tincture of cantharides. The facts in our possession point to the volatile principle in the living flies, which give them their disagreeable odor, as that which most rapidly occasions sexual excitement.

